# Bleomycin Concentration in Patients’ Plasma and Tumors after Electrochemotherapy. A Study from InspECT Group

**DOI:** 10.3390/pharmaceutics13091324

**Published:** 2021-08-24

**Authors:** Ales Groselj, Masa Bosnjak, Mojca Krzan, Tina Kosjek, Kriszta Bottyán, Helena Plesnik, Crt Jamsek, Maja Cemazar, Erika Kis, Gregor Sersa

**Affiliations:** 1 Department of Otorhinolaryngology and Cervicofacial Surgery, University Medical Centre Ljubljana, Zaloska 2, SI-1000 Ljubljana, Slovenia; ales.groselj@kclj.si (A.G.); crt.jamsek@kclj.si (C.J.); 2 Faculty of Medicine, University of Ljubljana, Vrazov trg 2, SI-1000 Ljubljana, Slovenia; 3 Department of Experimental Oncology, Institute of Oncology Ljubljana, Zaloska 2, SI-1000 Ljubljana, Slovenia; mbosnjak@onko-i.si (M.B.); mcemazar@onko-i.si (M.C.); 4 Faculty of Pharmacy, University of Ljubljana, Aškerčeva 7, SI-1000 Ljubljana, Slovenia; 5 Department of Pharmacology and Experimental Toxicology, Faculty of Medicine, University of Ljubljana, Korytkova 2, SI-1000 Ljubljana, Slovenia; mojca.limpel@mf.uni-lj.si; 6 Department of Environmental Sciences, Jozef Stefan Institute, Jamova 39, SI-1000 Ljubljana, Slovenia; tina.kosjek@ijs.si (T.K.); helena.plesnik@ijs.si (H.P.); 7 Jozef Stefan International Postgraduate School, Jamova 39, SI-1000 Ljubljana, Slovenia; 8 Department of Dermatology and Allergology, University of Szeged, 6721 Szeged, Hungary; krisztabottyan@gmail.com; 9 Faculty of Health Sciences, University of Primorska, Polje 42, SI-6310 Izola, Slovenia; 10 Faculty of Health Sciences, University of Ljubljana, Zdravstvena pot 5, SI-1000 Ljubljana, Slovenia

**Keywords:** electrochemotherapy, electroporation, bleomycin, pharmacokinetics, blood samples, tumor samples, drug delivery

## Abstract

The plasma concentration profile of bleomycin in the distribution phase of patients younger than 65 years is needed to determine the suitable time interval for efficient application of electric pulses during electrochemotherapy. Additionally, bleomycin concentrations in the treated tumors for effective tumor response are not known. In this study, the pharmacokinetic profile of bleomycin in the distribution phase in 12 patients younger than 65 years was determined. In 17 patients, the intratumoral bleomycin concentration was determined before the application of electric pulses. In younger patients, the pharmacokinetics of intravenously injected bleomycin demonstrated a faster plasma clearance rate than that in patients older than 65 years. This outcome might indicate that the lowering of the standard bleomycin dose of 15,000 IU/m^2^ with intravenous bleomycin injection for electrochemotherapy is not recommended in younger patients. Based on the plasma concentration data gathered, a time interval for electrochemotherapy of 5–15 min after bleomycin injection was determined. The median bleomycin concentration in tumors 8 min after bleomycin injection, at the time of electroporation, was 170 ng/g. Based on collected data, the reduction of the bleomycin dose is not recommended in younger patients; however, a shortened time interval for application of electric pulses in electrochemotherapy to 5–15 min after intravenous bleomycin injection should be considered.

## 1. Introduction

Electrochemotherapy is a local cancer ablative therapy that is currently performed in numerous cancer centers throughout Europe. Drug delivery into cells in electrochemotherapy is based on a physical delivery method called electroporation. Tumor tissue is exposed to an external electric field and delivered through electrodes with different geometries that correspond to different sizes and shapes of the treated tumors. Different malignancies, such as melanoma, basal cell carcinoma, cutaneous squamous cell carcinoma, breast cancer, Kaposi sarcomas, and others, are effectively treated with electrochemotherapy. The objective response rate of skin tumors is approximately 80% [1,2,3,4]. Nevertheless, electrochemotherapy has been shown to have different effectiveness in tumors of different histotypes; basal cell carcinomas have proven to be the most responsive, with an objective response rate of up to 95%, while melanomas have an objective response rate of approximately 70%. Studies have demonstrated that the response to electrochemotherapy is also dependent on the size of the tumors and eventual previous treatment(s) either with chemotherapy, radiotherapy, or surgery [5]. The most recent analysis of electrochemotherapy effectiveness in cutaneous malignancies in 987 patients with 2482 treated lesions was published in 2020 [6]. For the smallest lesions (less than 5 mm), the analysis showed 82% complete and 91% objective response rates after a single therapy. Not only cutaneous lesions but also deep-seated tumors, such as hepatocellular carcinoma or liver metastases of colorectal carcinoma, could be effectively treated with electrochemotherapy, and different response rates were observed, with hepatocellular carcinoma being more responsive [7,8].

The underlying mechanisms of therapeutic effectiveness have not yet been clearly elucidated. A preclinical search for the biological markers of tumor response to electrochemotherapy has already been performed. Intrinsic sensitivity to chemotherapeutic drugs, the immunophenotype of tumors and immunocompetence of the host, and vascularization of tumors partially determine the response to electrochemotherapy [9,10,11,12]. In addition, BRAF mutation was found to be a biological marker of synergistic effects with BRAF inhibitors and electrochemotherapy [13]. Olaparib, which is a DNA repair inhibitor, and electrochemotherapy have synergistic effects in *BRCA*-mutated breast cancer cells [14]. Viral infection, such as HPV infection, can also predispose tumor cells to a better response to electrochemotherapy [15].

For better planning and performing clinical studies using electrochemotherapy, a research roadmap for the investigation of biological factors of tumor response to electrochemotherapy has been published [16]. One of the predictive factors could also be bleomycin concentrations in plasma and tumors. The pharmacokinetics of bleomycin in patients has been defined by radioimmunoassay and high-pressure liquid chromatography but not by liquid chromatography mass spectrometry [17,18]. The first report of bleomycin pharmacokinetics in patients older than 65 years treated with electrochemotherapy was published by our group in 2016 [19] after the method for detection of bleomycin in serum and tumor samples was established [20]. The results demonstrated that the bleomycin dose in elderly patients can be reduced by one-third. No significant difference in long-term disease-free survival was observed in elderly patients treated with a reduced dose of bleomycin compared to those treated with a standard dose of bleomycin [21]. We also observed less intensive post electrotherapy reactions, shorter healing times, and better cosmetic outcomes in elderly patients treated with a reduced dose of bleomycin [22]. Other groups have also demonstrated a good antitumor response after reducing the bleomycin dose in elderly patients [23].

Based on the mentioned studies, we formulated one of our main questions as follows: what is the time course of bleomycin plasma concentration in patients younger than 65? Can we reduce the dose of bleomycin also in this age group, or is the time course of bleomycin plasma concentration profile significantly different from the 65 years old and older patients that were already published? In relation to the pharmacokinetic profile in patients younger than 65 years, the time interval for electrochemotherapy, i.e., time between the intravenous bleomycin injection and application of electric pulses, was compared to that of the older patient population.

Moreover, the concentrations of bleomycin in tumors have not yet been determined. Therefore, in this study, we wanted to determine the correlation between concentrations of bleomycin in tumors and response to the treatment. Furthermore, we also wanted to determine the minimal effective dose in tumors needed for a good antitumor response.

Altogether, we aimed to determine bleomycin pharmacokinetic aspects upon a physically mediated drug delivery method, electroporation, which would serve us to better understand the efficacy and safety aspects of electrochemotherapy and its application in younger and older patients’ populations.

## 2. Patients and Methods

### 2.1. Patients

The study was conducted as a prospective, interventional study between October 2017 and December 2019 within the International Network for Sharing Practices of electrochemotherapy (InspECT) (www.insp-ect.org, accessed 15 March 2021). All patients who presented with skin cancers, recurrent disease (and/or metastatic disease) or a second primary tumor in the head and neck region were either not suitable for conventional treatments or refused them. The patients were treated with electrochemotherapy at the Department of Otorhinolaryngology and Cervicofacial Surgery, University Medical Centre Ljubljana, Slovenia or the Department of Dermatology and Allergology, University of Szeged, Hungary. The study was approved by the National Ethics Committee of the Republic of Slovenia (0120-296/2017-3, 25 March 2017) and the Regional and Institutional Review Board of Human Investigations at the University of Szeged, Hungary (4129-223/2017, 20 November 2017). The study was registered in the ISRCTN database (ISRCTN74054653). Treatment with electrochemotherapy was previously agreed upon by a multidisciplinary board, and written informed consent was obtained from all eligible patients. The selection of patients was performed according to the NICE guidelines. The included patients were older than 18 years and presented with different histologies of cutaneous tumors (basal cell carcinoma, squamous cell carcinoma, melanoma, and others). The exclusion criteria were as follows: (a) patients not suitable for electrochemotherapy according to the NICE guidelines and (b) patients who did not comply with requirements for blood collection, tumor biopsy or tissue sampling according to the SOP for blood collection or tissue sampling. In the study, altogether 25 patients were included; 8 patients younger than 65 for blood sample collection, 4 patients younger than 65 for blood and tumor sample collection and 13 patients for tumor sample collection with no age limitation.

The study was conducted as a prospective, interventional study between October 2017 and December 2019 within the International Network for Sharing Practices of electrochemotherapy (InspECT) (www.insp-ect.org, accessed 15 March 2021). All patients who presented with skin cancers, recurrent disease (and/or metastatic disease) or a second primary tumor in the head and neck region were either not suitable for conventional treatments or refused them. The patients were treated with electrochemotherapy at the Department of Otorhinolaryngology and Cervicofacial Surgery, University Medical Centre Ljubljana, Slovenia or the Department of Dermatology and Allergology, University of Szeged, Hungary. The study was approved by the National Ethics Committee of the Republic of Slovenia (0120-296/2017-3, 25 March 2017) and the Regional and Institutional Review Board of Human Investigations at the University of Szeged, Hungary (4129-223/2017, 20 November 2017). The study was registered in the ISRCTN database (ISRCTN74054653). Treatment with electrochemotherapy was previously agreed upon by a multidisciplinary board, and written informed consent was obtained from all eligible patients. The selection of patients was performed according to the NICE guidelines. The included patients were older than 18 years and presented with different histologies of cutaneous tumors (basal cell carcinoma, squamous cell carcinoma, melanoma, and others). The exclusion criteria were as follows: (a) patients not suitable for electrochemotherapy according to the NICE guidelines and (b) patients who did not comply with requirements for blood collection, tumor biopsy or tissue sampling according to the SOP for blood collection or tissue sampling. In the study, altogether 25 patients were included; 8 patients younger than 65 for blood sample collection, 4 patients younger than 65 for blood and tumor sample collection and 13 patients for tumor sample collection with no age limitation.

### 2.2. Procedure

Electrochemotherapy was performed according to the updated standard operating procedures [24]. Briefly, bleomycin (bleomycin medac, Medac, Wedel, Germany) was injected intravenously (bolus in 2 min) at a dose of 15,000 IU/m^2^ body surface area (1000 IU is equal to 1 mg of bleomycin activity), and 8 min after the injection, electric pulses were applied. Electric pulses for electrochemotherapy were generated by a Cliniporator pulse generator (IGEA, s.r.l., Carpi, Italy) and delivered by fixed geometry electrodes, i.e., mainly electrodes with eight needles arranged in two rows of four needles each with a distance of 4 mm between the rows. In one application of electric pulses, eight pulses with a voltage of 400 V, a pulse duration of 100 µs and a frequency of 5000 Hz were applied. The number of applications was adjusted to the size of the treated tumors.

### 2.3. Blood Samples Collection

For blood collection, 12 patients younger than 65 years were eligible. Two milliliters of arterial blood samples were collected at predetermined time points (0, 5, 10, 20, 30, and 40 min after bleomycin administration, where 0 is considered the time immediately before bleomycin injection) into blood collection tubes (BD Vacutainer, Franklin Lakes, NJ, USA). Samples were centrifuged at 1882× *g* for 10 min at room temperature. Plasma was carefully transferred to a new Eppendorf tube and stored at −20 °C until analysis.

### 2.4. Tumor Samples Collection

For tumor tissue collection, 17 patients either younger or older than 65 years (4 were the same patients as for blood collection) were eligible. Eligible patients should have had enough tumor volume for 3-mm punch biopsy and further electrochemotherapy procedures. A one-time punch biopsy was performed just before application of electric pulses, 8 min after intravenous bleomycin injection. Multiple biopsies were not taken because of relatively small tumor volumes. The tumor samples were taken from the visibly viable tumor section, and the sample was taken throughout the tumor thickness. The samples were placed into cryotubes (Corning Incorporated, Corning, NY, USA) and stored at −80 °C until further analysis.

### 2.5. Chemical Analysis

Plasma samples were prepared for analysis by protein precipitation and phospholipid removal using Ostro™ 96-well plates (Waters Corp., Milford, MA, USA). A 200-µL plasma sample was added to the 2-mL well, followed by 600 µL acetonitrile with 0.1% formic acid and internal standard epirubicin at a final concentration of 0.05 µg/mL. The mixture was then aspirated three times with a pipette and pushed through the Ostro^TM^ sorbent at 60 psi N_2_ for 5 min. The extract was finally filtered through a 0.2-µm regenerated cellulose filter and analyzed by liquid chromatography coupled to tandem mass spectrometry (LC-MS/MS).

Tumor samples were ground to a fine powder under liquid nitrogen, suspended in 6 mL 0.1% HCOOH, sonicated for 60 min, centrifuged at 12,900 RCF for 20 min and filtered through 0.45-µm cellulose acetate filters before solid-phase extraction using Oasis HLB 1 cc/30 mg cartridges. After the sample filtrates were loaded onto the cartridges, the sorbent was dried under vacuum for 30 min and subsequently eluted using 0.5 mL of Milli-Q water/methanol (6/4) twice with 0.5 mL acetonitrile solution. As follows, the sample extracts were blown down to 1 mL using N_2_ and were then kept frozen until LC-MS/MS analysis, which was performed within one month.

LC-MS/MS analysis was performed with a Nexera ultrahigh-performance LC (Shimadzu Corp., Kyoto, Japan) coupled to a QTRAP^®^ 4500 MS/MS system (AB Sciex, Framingham, MA, USA). Separation was achieved at room temperature using a 5-cm-long Acquity UPLC BEH Amide (Waters Corp., Milford, MA, USA) column with a 1.7-µm particle size and 2.1-mm internal diameter. The mobile phases were 10 mM ammonium formate with 0.1% formic acid and acetonitrile. The flow rate was 0.3 mL/min. The mass spectrometer was operated in positive electrospray ionization with the multiple reaction monitoring acquisition mode. The data station operating software used was Analyst^®^ v1.6.3 (AB Sciex, Framingham, MA, USA).

The method linearity range was 0.006 μg/mL to 1.5 μg/mL at R^2^ of 0.995. The method detection limit was 0.045 μg/mL and the quantification limit 0.14 μg/mL. The intraday variation was 12% at low (0.02 μg/mL) and 2% at medium (0.60 μg/mL) BLM levels. The interday variations were 19% and 16% for low and medium levels, respectively.

### 2.6. Pharmacokinetic Data Analysis

Bleomycin plasma concentrations versus the time of each patient and the combined data of all 12 patients were analyzed. We determined the following pharmacokinetic parameters: the area under the plasma concentration-time curve (*AUC*), which was calculated using the trapezoid method from time 0 to 40 min after single bolus intravenous injection (*AUC* 0 → 40), and plasma clearance (*Cl*) was calculated as follows:

(1) $$Cl=\frac{dose}{AUC\;0\to 40}$$

The elimination rate constant *k_el_* was determined from the slope of ln (concentration) versus the time curve, and half-life was calculated using the following:

(2) $$t\;½=\frac{\ln2}{k_{el}}$$

and volume of distribution (*Vd*)

(3) $$Vd=\frac{t\;½}{\ln2}\times Cl$$

### 2.7. Tumor Response Analysis

The tumor volumes were calculated according to the SOP, i.e., a × b^2^ × π/6. Tumor response 2 months after electrochemotherapy was evaluated with a clinical exam according to RECIST 1.1 criteria [25].

### 2.8. Statistical Analysis

The values in this study are represented as arithmetic mean (AM) ± standard error of the mean (SE) unless otherwise stated. Comparisons between two groups were performed using unpaired 2-tailed Student’s *t*-test. A *p*-value of less than 0.05 was considered to be statistically significant. GraphPad Prism (GraphPad, San Diego, CA, USA) was used for statistical analysis and graphical representation. 

## 3. Results

### 3.1. Bleomycin Pharmacokinetics in Plasma of Younger Patients

The time course of the plasma concentration of bleomycin was determined in 12 patients younger than 65 years. The patients were treated with a standard dose of bleomycin, i.e., 15,000 IU/m^2^ bleomycin in bolus injection. The patients’ demographics are presented in Table 1, and their median age was 52.2 years, with a range from 35 to 64. Patients presented with cutaneous tumors eligible for electrochemotherapy. The predominant tumor types were SCC (*n* = 6) and BCC (*n* = 5). The median dose of bleomycin used in electrochemotherapy was 27,404 ± 2637 IU. In all, except for three patients who were either off study or lost to follow-up, the complete response (CR) of the treated tumors was achieved.

**Figure 1:**
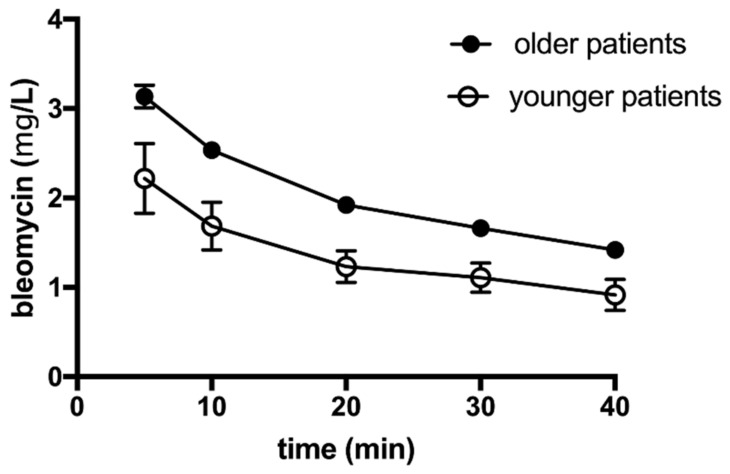
The elimination of bleomycin from the plasma of patients younger and older than 65 years after intravenous bolus injection of 15,000 IU/m^2^ bleomycin dose. The points represent the mean +/− SEM value determined in 12 patients. The graph for older patients has already been published, but due to direct comparison, we present the data on the same graph (with permission).

Blood samples were drawn at several time points, i.e., 5, 10, 20, 30, and 40 min after bleomycin intravenous injection, and bleomycin concentration was determined in the plasma of these patients. Bleomycin concentrations were determined using liquid chromatography coupled to tandem mass spectrometry. Based on the measurements, the pharmacokinetic parameters of bleomycin elimination were determined. The plasma concentration-time profile following intravenous bolus injection of bleomycin is shown in Figure 1. For comparison, the same graph shows the pharmacokinetic profile of plasma concentration-time profile in the patients who were older than 65 years, which is information taken from our previous publication [19].

In patients younger than 65 years, following a single bolus intravenous injection of bleomycin (24,000–30,000 IU, mean dose 27,404 ± 2637 IU) plasma concentration 5 min after injection was 2.219 ± 0.391 mg/L. The concentration of bleomycin declined gradually with an elimination constant (*k_el_*) of 0.068 ± 0.059 min^−1^ and reached a value of 0.915 ± 0.173 mg/L at 40 min after injection (Figure 1).

The apparent volume of distribution (*Vd*), area under the curve (*AUC*), plasma clearance (*Cl*) and elimination half-life of bleomycin were calculated and are shown in Table 2. In the same table are also the pharmacokinetic parameters of the previously published data on patients older than 65 years, which were determined and calculated the same way as in this study [19].

Although the half-life of bleomycin was not significantly different from that in older patients, significant differences were observed in AUC, plasma clearance and volume of distribution. The AUC in younger patients was significantly lower than that in older patients, which is consistent with significantly increased plasma clearance in younger patients compared to older patients. In addition to increased clearance, the increased volume of distribution of bleomycin was determined in younger patients in comparison with older patients. A possible explanation for these two observations is that older patients have age-related decreases in kidney function and different body compositions regarding the water/fat ratio. Therefore, these results indicate that the bleomycin dose in patients younger than 65 years could not be decreased unless their kidney function was impaired.

The ideal time interval for electrochemotherapy in older patients was determined at 8–40 min after intravenous bleomycin bolus injection. From the elimination curves in this study, it was calculated that the plasma clearance of bleomycin in younger patients is much more abundant than the plasma clearance of bleomycin in older patients. Specifically, the plasma concentration that has been determined in older patients at 40 min after bleomycin injection (trough concentration) has already been reached at 15 min after bleomycin injection in younger patients. Moreover, the peak plasma concentration was reached in 5 min in younger patients compared to 8 min after bleomycin injection in older patients. We can conclude that the ideal time interval for electrochemotherapy being at peak-trough levels of bleomycin in systemic circulation lies between 5 and 15 min in younger patients and between 8 and 40 min in older patients.

### 3.2. Bleomycin Dose Distribution in Tumor Samples

Tumor tissue was obtained with punch tumor biopsies of tumors treated by electrochemotherapy from 17 patients regardless of the age at the time of electrochemotherapy. Patients were predominantly older than 65 years (*n* = 12), and the median age of the patients was 72.8 years (range = 49–85 years). All patients were treated with electrochemotherapy using 15,000 IU/m^2^ intravenous bleomycin bolus injection, and biopsies were taken from one tumor per patient 8 min after bleomycin administration, just before the application of electric pulses (Table 3).

**Figure 2:**
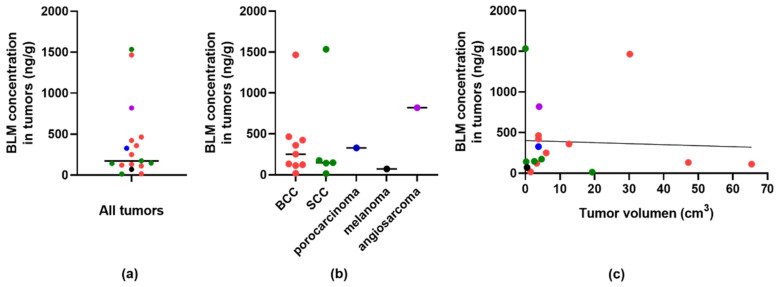
Median bleomycin concentrations in tumor samples. (**a**) Bleomycin (BLM) concentrations normalized to 1 g of the tumor sample in all analyzed tumors. (**b**) Bleomycin (BLM) concentrations normalized to 1 g of the tumor sample in correlation to tumor type. (**c**) Correlation of bleomycin (BLM) concentrations in tumor and tumor volume with linear curve fit analysis.

The predominant samples were biopsies from BCC (*n* = 9) and SCC (*n* = 5) and one sample each from porocarcinoma, melanoma, and angiosarcoma. The bleomycin concentrations determined ranged from 14 ng/g of tumor to 1534 ng/g of tumor (Figure 2a). The median concentration was 174 ng/g of tumor, regardless of tumor histology. No significant differences in bleomycin concentration between the BCC and SCC were determined (*p* = 0.917) (Figure 2b). The tumor complete response rate 2 months after therapy was 89% (8/9) in BCC and 80% (4/5) in SCC. There was no correlation between the volume of the tumor and the concentration determined in the punch biopsy sample (Figure 2c).

## 4. Discussion

This study is the first to determine the pharmacokinetic profile of bleomycin in the distribution phase after intravenous bolus injection in patients younger than 65 years. The bleomycin was eliminated from plasma with an elimination constant of 0.068 min^−1^. The study also provides preliminary data about the minimal bleomycin concentration in tumors of 14–18 ng/g to induce complete responses of electrochemotherapy-treated tumors. Furthermore, the data also indicate that there is no significant difference in bleomycin tumor concentration between BCC and SCC tumors and no correlation between intratumoral bleomycin concentration and tumor size. Based on the plasma concentration data, a time interval for electrochemotherapy of 5–15 min after intravenous bolus bleomycin injection is recommended for patients younger than 65.

This is a preliminary study due to its limitations, such as the low number of included patients. The data on younger patients were compared to the data collected in previous study on older patients, but still within the same clinical study. This was due to slow recruitment of younger patients. Another obstacle was that in some patients, blood and tumor tissue collection was not possible, due to their physical condition or tumor size or location. To overcome these drawbacks of the study a broader multicentric study is needed as well to obtain more data on bleomycin concentrations in tumors and correlate them with tumor size and tumor type.

Our previous study determined the effective time interval for electrochemotherapy of 8–40 min after bleomycin administration in older patients [19]. The slow elimination rate of bleomycin from systemic circulation in older patients implies slow bleomycin elimination due to the physiological specificities of the older population and age-related decline in kidney function. In younger patients, i.e., less than 65 years of age, bleomycin is eliminated faster than in elderly patients. These differences in plasma concentration and elimination rate are important, since they indicate that the physiological parameters related to patient age need to be considered to ensure safe and effective treatment of electrochemotherapy using bleomycin that is given intravenously.

Can we conclude that lowering the therapeutic dose of bleomycin in electrochemotherapy in patients younger than 65 years is not indicated? The data provided in this manuscript suggest that lowering the dose might not be recommended. However, a correlation between the age and pharmacokinetics of bleomycin in different age groups is needed to confirm the indication for a lower dose. Furthermore, we would need to have a correlation between the plasma bleomycin concentration and tumor concentration, which our study could not demonstrate. Only three patients had both data points, which is not sufficient to draw any conclusions. In the standard operating procedures, it is also stated that lowering the dose is indicated upon demonstration of impaired kidney function. Creatinine levels might not be the sole indicator of kidney impairment, especially in the elderly population, where production of creatinine is decreased due to age-related reduction in skeletal muscle mass and there would also be a need for other factors upon which the indications of lowering the dose are needed [19]. Furthermore, the study of Rotunno et al. demonstrated that lowering the dose by more than 30% could be effective in electrochemotherapy [23]. Therefore, further studies are needed to explore this possibility more in depth. Nevertheless, this study provides preliminary data on bleomycin pharmacokinetics for treatment with electrochemotherapy using intravenously administered bleomycin, and further studies can build upon these data.

Intratumoral bleomycin determination in patients treated with electrochemotherapy has not yet been performed, except for murine tumors [11]. In electrochemotherapy the bleomycin dose is so low that without electroporation it does not have antitumor effectiveness. This was demonstrated in preclinical studies and in early clinical trials as well [26,27]. To study in more detail pharmacokinetics of bleomycin in electrochemotherapy we used recently developed liquid chromatography coupled to tandem mass spectrometry method to explore the following three questions [20]. What is the minimal drug concentration in tumors for the complete response of tumors? Is there a difference in drug concentration between different tumor types? Is there a correlation between the tumor size and drug concentration? Based on the small amount of data, all three questions were partially answered. In this study, we had 17 tumor samples of predominantly BCC and SCC tumors. We determined the approximate tumor concentration of bleomycin in these two tumor types, with a median concentration of 174 ng/g of tumor. In these two tumor types, a minimal bleomycin concentration of 14–18 ng/g of tumor was needed to provide the complete response of the tumors to electrochemotherapy. However, BCC at 133 ng/g responded only partially to electrochemotherapy. Thus, contrary to the results obtained in murine tumors where the correlation between intratumoral bleomycin concentration at the time of electroporation and antitumor effectiveness was obtained, this was not confirmed in clinical samples. Further preclinical and clinical studies are needed to determine the parameters that affect the accumulation and distribution of drugs in tumors. Additionally, we did not show a correlation between bleomycin concentration in the tumor and tumor size. The concentration of bleomycin is most likely dependent on the site of punch biopsy sampling and vascularization of the tumors. Interestingly, in angiosarcoma, where the bleomycin concentration was among the highest, the tumor progressed while not responding to electrochemotherapy. This observation might be explained by the differences in vascularization and perfusion in different histological types of tumors.

In this study, we evaluated the bleomycin concentration in tumors after intravenous drug administration. However, studies on drug accumulation in tumors after intratumoral administration are needed. The drug concentration and drug distribution might be different from intravenous administration. Intratumoral drug administration is quite rare in clinical studies; however, with more evidence, it might be more frequently used. A recent study in a senior population showed that older patients can also be treated with electrochemotherapy, and in such cases, local anesthesia and intratumoral drug administration are recommended [16,22]. Other studies that address the drug distribution within tumors are also needed. To date, a suitable method has been developed for the distribution of cisplatin [28]. In that study, high spatial resolution imaging of cisplatin distribution in spheroids of tumor cells was determined using two different approaches, namely, fluorescently labeled cisplatin determined by confocal fluorescence microscopy and laser ablation isotope dilution inductively coupled plasma mass spectrometry. The results demonstrated satisfactory agreement in terms of the spatial distribution between the two methods, and the intensity of the fluorescence matched well with the concentrations of platinum obtained by laser ablation isotope dilution inductively coupled plasma mass spectrometry; thus, this technique could be used reliably for human tumor samples. Therefore, studies comparing the pharmacodynamics of bleomycin between intravenous and intratumoral administration are needed. The needed concentrations of the drug for good antitumor effectiveness would enable better treatment approaches.

## 5. Conclusions

Lower plasma concentrations of bleomycin and faster elimination rate of bleomycin in patients younger than 65 years treated with electrochemotherapy than in patients older than 65 years might indicate that lowering the standard bleomycin dose of 15,000 IU intravenous bleomycin injection for electrochemotherapy is not recommended in younger patients. Based on the bleomycin plasma concentration data gathered in this study, the suggested time interval for electrochemotherapy is 5–15 min after the intravenous bleomycin injection for patients younger than 65 years.

## Figures and Tables

**Table 1:** Characteristics of patients eligible for blood collection, according to the tumor histotype, initial tumor volume, age and bleomycin dose given in treatment with electrochemotherapy and tumor response after 2 months according to RECIST 1.1 criteria.

Patient No.	Gender	Age at ECT (Years)	Localization	Histology	Number of Treated Lesions	Bleomycin Dose (IU)	Response at 2 Months	Tumor Volume (cm^3^)
1	M	45	Skin	SCC	1	30,000	CR	0.6
2	M	59	Skin	SCC	1	24,000	Lost to FU	20.5
3	M	35	Skin	BCC	1	30,000	PR	17.7
4	F	39	Skin	BCC	1	24,000	CR	3.5
5	M	49	Skin	SCC	4	30,000	CR	0.3, 14.1, 12.8, 6.3
6 *	M	58	Skin	SCC	6	30,000	CR	30.0, 5.6, 0.4, 0.2, 0.2, 19.3
7 *	M	49	Skin	BCC	1	26,000	CR	3.8
8	M	59	Skin	SCC	1	30,000	CR	5.2
9	F	50	Skin	BCC	1	25,000	CR	10.6
10	F	55	Skin	BCC	1	26,000	Lost to FU	0.2
11 *	M	63	Skin	Melanoma	3	29,100	CR	0.5, 1.0, 3.8
12 *	M	64	Skin	SCC	7	24,750	Off study	0.2, 0.6, 0.8, 0.7, 0.6, 0.9, 0.8
Total = 12	3 F/9 M	Median = 52.2 Range = 35–64				Mean dose = 27,404 ± 2637		

BCC—basal cell carcinoma, CR—complete response, FU—follow-up, PR—partial response, SCC—squamous cell carcinoma, * patients also eligible for tumor sample collection.

**Table 2:** Comparison of pharmacokinetic parameters, obtained in 40 min post-bleomycin intravenous injection, of patients younger and older than 65 years treated by electrochemotherapy with 15,000 IU/m^2^ bleomycin.

Sub-Groups of Patients	Number of Patients	*t*_1/2_ (min)		*AUC* 0 → 40 (µg min/mL)		Plasma Clearance *Cl* (mL/min)		*Vd* (L)	
**Younger Patients**	12								
AVG		24.84	n.s.	50.83	*p* < 0.0001	999.1	*p* = 0.0007	32.17	*p* = 0.0083
SEM		2.10		7.90		261.0		6.24	
Lower 95% CI of mean		20.22		33.44		424.6		18.44	
Upper 95% CI of mean		29.46		68.22		1574.0		45.90	
**Older Patients**	24								
AVG		29.4		86.02		311.3		15.9	
SEM		1.79		7.899		15.83		2.65	
Lower 95% CI of mean		25.69		79.55		278.6		10.42	
Upper 95% CI of mean		33.10		92.49		344.1		21.37	

n.s.—not statistically significant.

**Table 3:** Characteristics of patients eligible for one tumor sample collection, according to the tumor histotype, age of patients, bleomycin dose given in treatment with electrochemotherapy and tumor response after 2 months according to RECIST 1.1 criteria.

Patient No.	Gender	Age at ECT (Years)	Localization	Histology	Number of Treated Lesions	Bleomycin Dose (IU)	Tumor Response (All Tumors)	Tumor Response (Tumor with Biopsy)	Bleomycin Concentration per 1 g of Tumor (ng/g)	Tumor Volume (cm^3^)
1	F	85	Skin	BCC	3	24,000	1 CR, 2 PR	PR	133	47.1
2	M	65	Skin	BCC	1	30,000	CR	CR	123	3.3
3	F	85	Skin	BCC	2	25,000	2 CR	CR	359	12.6
4 *	M	49	Skin	BCC	1	26,000	CR	CR	464	3.8
5	M	81	Skin	BCC	5	27,000	4 CR, 1 SD	CR	250	6.0
6	F	82	Skin	BCC	1	24,000	CR	CR	112	65.4
7	F	76	Skin	BCC	5	24,000	5 CR	CR	18	1.5
8	F	79	Skin	BCC	1	29,850	CR	CR	422	3.8
19	F	78	Skin	BCC	16	21,900	11 CR, 5 PR	CR	1466	30.2
10	M	76	Skin	SCC	1	27,000	CR	CR	147	2.6
11 *	M	58	Skin	SCC	6	30,000	6 CR	CR	14	19.3
12	M	85	Skin	SCC	1	24,000	CR	CR	174	4.7
13	M	62	Skin	SCC	1	26,000	CR	CR	142	0.2
14 *	M	64	Skin	SCC	7	24,750	Off study	/	1534	0.2
15 *	M	63	Skin	Melanoma	3	29,100	3 CR	CR	71	0.5
16	F	70	Skin	Angiosarcoma	1	28,650	PD	PD	819	4.0
17	M	81	Skin	Porocarcinoma	2	29,850	2 CR	CR	327	3.8
Total = 19	7 F/10 M	Median = 72.8 Range = 49–85				Mean dose = 26,535 ± 2631			Median = 174 Range = 14–1534	

BCC—basal cell carcinoma, CR—complete response, PR—partial response, SCC, squamous cell carcinoma, SD—stable disease, PD—progressive disease, * patients also eligible for blood sample collection.

## Data Availability

The data presented in this study are available on request from the corresponding author. The data are not publicly available due to the privacy of the patients.
